# Wind Data Mining by Kohonen Neural Networks

**DOI:** 10.1371/journal.pone.0000210

**Published:** 2007-02-14

**Authors:** José Fayos, Carolina Fayos

**Affiliations:** 1 Departamento de Cristalografía, Instituto de Química Física Rocasolano, Consejo Superior de Investigaciones Científicas (CSIC), Madrid, Spain; 2 Raytheon Information Solutions, Silver Spring, Maryland, United States of America; Purdue University, United States of America

## Abstract

Time series of Circulation Weather Type (CWT), including daily averaged wind direction and vorticity, are self-classified by similarity using Kohonen Neural Networks (KNN). It is shown that KNN is able to map by similarity all 7300 five-day CWT sequences during the period of 1975–94, in London, United Kingdom. It gives, as a first result, the most probable wind sequences preceding each one of the 27 CWT Lamb classes in that period. Inversely, as a second result, the observed diffuse correlation between both five-day CWT sequences and the CWT of the 6^th^ day, in the long 20-year period, can be generalized to predict the last from the previous CWT sequence in a different test period, like 1995, as both time series are similar. Although the average prediction error is comparable to that obtained by forecasting standard methods, the KNN approach gives complementary results, as they depend only on an objective classification of observed CWT data, without any model assumption. The 27 CWT of the Lamb Catalogue were coded with binary three-dimensional vectors, pointing to faces, edges and vertex of a “wind-cube,” so that similar CWT vectors were close.

## Introduction

The current Circulation Weather Type (CWT), including wind direction and vorticity, gives local information about the upcoming temperature and precipitation, as it is shown by Hulme et al. [1]. Besides, CWT prediction is also important for practical uses such as aerial and water transport. However, the nonlinear behaviour of the dynamical phenomena involving these variables adds difficulty to dynamical models to predict CWT. Thus, having into account the large amount of available CWT observed data, a data mining approach seems to be appropriate to uncover correlations among time series of CWT, not easily expressed in the model equations. Data mining results depend just on the observed CWT behaviour along large periods, where time series seem to repeat somehow allowing prediction. Hence, being independent of any behaviour model assumed for CWT, data mining can also be complementary information for the well established forecasting methods.

Neural Networks (NN) have proved to be an efficient tool for weather data mining. For example Kretzschmar et al. [2] and Marzban [3] worked on the local prediction of the wind speed by using feed-forward NN. Marzban [3] selected wind direction as a predictor and their results, especially on predicting anomalous winds, improved somewhat to those obtained by other methods such as linear regression or persistence. Both authors show that wind persistence is indeed quite frequent and therefore it should be a reference for any other prediction method.

In the present work, we used the CWT Lamb Catalogue with the averaged daily wind direction and vorticity over London (UK) area, Lamb [4], [5]. The Catalogue describes the state of the large-scale atmospheric circulation, from 1861 to 1997. The CWT data are calculated from a Northern Europe grid, centred in London, at sea-level and 500mbar pressure, taken twice daily at 00GMT and at 12GMT. These data are available on the web: http://www.cru.uea.ac.uk/∼mikeh/datasets/uk/lamb.htm. The Catalogue was recalculated to an objective one by Jenkinson and Collison [6] and then used by Jones et al. [7]. Later, Cawley and Dorling [8] reported a neural network classifier to reproduce the Lamb classification using the Jenkinson algorithm. Research about the calculation and discussion of this catalogue, are shown by Briffa [9] and Chen [10], the latter also describing the calculation of the wind direction derived from the pressure grid. The Lamb Catalogue has been used in some studies, Davis et al. [11], relating atmospheric circulation to environmental problems.

These CWT data, for a long (20 years) learning period, were used in this work for both predictor and predictant, where short (five days) time series of daily CWT sequences were classified by their resultant wind of the next day. It is shown in fact that both sets are correlated. This classification produces an accumulative table with the most probable CWT sequences preceding each one of the 27 possible CWT Lamb classes, which can be generalized to another test period, to predict the unknown CWT from its observed (five days) precedent CWT sequence. Having into account the nonlinear behaviour of CWT variables, we select as an objective CWT classifier the Kohonen Neural Network (KNN), Kohonen [12], because it is a self-organizing feature map. It means that the KNN procedure would uncover some implicit CWT relations instead to assume any CWT behaviour. This is not the first time that KNN is used to weather forecasting, a detailed paper concerning its use for predicting one variable among time series of sea levels, wind data, air pressure and air temperature in the German North Sea is due to Ultsch and Röske [13]. They concluded that the implicit modelling of the physical processes employed in the NN predicts better than the explicit modelling employed in the hydrodynamic or statistical methods, although in fact their results gave higher errors for predicted wind direction.

## Results

### CWT data analysis

The Lamb Catalogue contains 27 Lamb Classes (LC) of daily averaged wind directions and vorticity, which are our CWT data. Table 1 shows the number of days per LC class that appear in the London Catalogue during 50 years (N = 18263 days) from 1947 to 1996, where null or undefined values can result after averaging. Notice that this is a highly inhomogeneous distribution of wind classes similar to the one given by Chen [10] for Sweden. This fact will obviously favor the prediction of the most frequent winds such as W against those least frequent such as CNE wind. The CWT distribution also shows many zero or neutral winds such as A or C, and many winds of W or SW directions (especially those with undefined circulation). However, as above mentioned, due to the frequent wind changes in the same day, both zero and undefined winds can be biased by their daily averaging, which can hide important daily variations. In any case, that lost information in the average CWT data, must be considered to interpret the predicted CWT also as daily average data.

**Table 1:** Distribution of the 27 LC wind classes in London, along 50 years.

A	ANE	AE	ASE	AS	ASW	AW	ANW	AN
3644	151	165	185	320	530	551	364	240
UND	NE	E	SE	S	SW	W	NW	N
198	323	326	497	1010	1641	1914	1076	657
C	CNE	CE	CSE	CS	CSW	CW	CNW	CN
2343	103	110	198	307	451	433	339	187

The first column is for null wind, the first and second rows are for anticyclone (A) vorticity, the third and fourth for undefined vorticity and the fifth and sixth for cyclone (C). The rest of acronyms are as conventional, N: North, S: South, E: East, W: West, and UND: total undefined for wind direction and vorticity.

We checked the above mentioned wind persistence by comparing the observed LC distribution with a random generated LC distribution during the 50 years period, assuming the frequencies of Table 1. For the random LC distribution, the probability of finding two equal LC in two randomly chosen days along a time series of N days with *n_i_* days per LC_i_ class is (∑*n_i_* ^2^)/*N* ^2^ = 0.09, which would give 1650 wind coincidences in N = 18263 random tests (independently of the separation between days). However, by using the actual observed wind distribution, we show in Table 2 that the number of wind coincidences is much greater than 1650 when the two selected days are separated by few day intervals, indicating a non-random wind distribution. We see that 29% of the days repeat the wind of the previous day (*interv* = 1), and in general wind persistence is observed for shorter periods. The number of wind coincidences decreases quickly from *interv* = 1 to 5 as shows the last row. Calculation up to *interv* = 40, shows that from 8 to 40 days intervals the average wind coincidences is 1700, which is similar to the above calculated value for a random wind distribution.

**Table 2:** LC wind coincidences (*coinc*) for two days separated by an interval of *interv* days, *δ(coinc)* is the difference between consecutive number of coincidences.

*interv*	10	9	8	7	6	5	4	3	2	1
*coinc*	1839	1864	1966	2072	2138	2322	2707	3059	3738	5330
*δ(coinc)*	25	102	106	66	184	385	352	679	1592	

Besides this explicitly observed wind persistence, we assume that some other wind information of the target day is implicit in the previous CWT time series, implicit correlation that will also be uncovered by KNN.

It is not easy to choose an optimal number of days for the sequence of daily winds previous to a target day, to have enough correlation in between. We see that the fewer days we take the more persistence effect will condition the correlation, but CWT of more distant days would not condition the target day. The *δ(coinc)* values of Table 2 show that five days seems to mark a wind persistence behaviour border, so we decided to take time series of five consecutive CWT to be correlated with the CWT of the 6^th^ day. Then, among the 50 year period, there is a 50% probability to have coincidence between the CWT of the 6^th^ day with any one of the five previous days.

### CWT data codification

**Figure 1:**
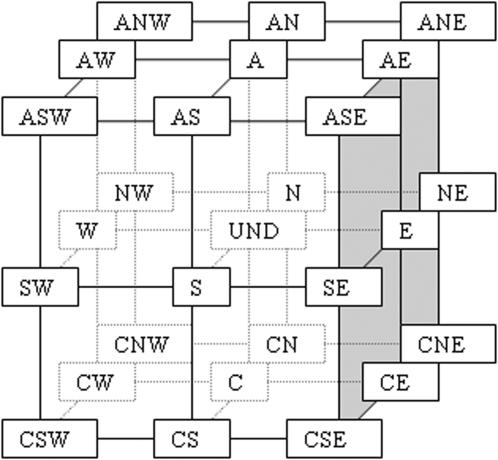
Winds cube representing the 27 Lamb Classes of CWT, including wind direction and vorticity, codified by 3D binary vectors BLC. The origin (000) represents the total UNDefined wind.

The KNN process compares, by similarity, the 27 LC's among them. But their orderly classification hinders such comparison, for example the LC for the wind ANE is 1 whereas LC for the wind AN is 8 and LC for the wind N is 18, although ANE and AN are near situations as well as AN and N are. In order to prevent this we codified each LC by a 3D binary vector (BLC), where the first component (1, 0 or −1) indicates its vorticity or circulation (A, UNDefined or C); the second component indicates the projection of the wind on the N-S direction: 1 for N-wind, 0 for no projection and −1 for S-wind; and the third component is the projection of the wind on the E-W direction: 1 for E-wind, 0 for no projection and −1 for W-wind. In this manner, the 27 3D vectors, shown in Table 3 (with the exception of the zero vector), point to the surface of a “wind's cube”, in vertex, face centres and edge centres, as Figure 1 shows, where close winds are represented by near points on the cube surface.

**Table 3:** Description of the 27 Lamb Codes and their 3D vector codifications used in this work.

0	A	(1 0 0)	10	UND	(0 0 0)	20	C	(−1 0 0)
1	ANE	(1 1 1)	11	NE	(0 1 1)	21	CNE	(−1 1 1)
2	AE	(1 0 1)	12	E	(0 0 1)	22	CE	(−1 0 1)
3	ASE	(1-1 1)	13	SE	(0-1 1)	23	CSE	(−1-1 1)
4	AS	(1-1 0)	14	S	(0-1 0)	24	CS	(−1-1 0)
5	ASW	(1-1-1)	15	SW	(0-1-1)	25	CSW	(−1-1-1)
6	AW	(1 0-1)	16	W	(0 0-1)	26	CW	(−1 0-1)
7	ANW	(1 1-1)	17	NW	(0 1-1)	27	CNW	(−1 1-1)
8	AN	(1 1 0)	18	N	(0 1 0)	28	CN	(−1 1 0)

The three groups, in columns, show the 9 classes (in rows) for anticyclone circulation (A), undefined circulation, and cyclone circulation (C). In each group the first column is the Lamb Code, the second column is the wind direction and the third column is our 3D vector codification (pointing to the surface of the winds cube shown in Figure 1). The classes of the first row correspond to zero daily average wind and in successive rows, rotating clockwise, are the eight conventional directions of the wind from North-East to North. The Lamb Code 10 is for winds of undetermined vorticity and direction.

For our KNN self-classification, we have coded the LC data in pairs of vectors, where the first vector represents the CWT of the 5 previous days (5LC) and the second vector represents the CWT of the 6^th^ day (LC6). The vector codifying 5LC is a (5x3)D vector (from now 5BLC) formed by the union of the five correspondent 3D vectors. The vector codifying LC6 is its 3D binary vector (BLC6). As an example, the LC's for the days 1 to 10 of January 1980 in the Lamb Catalogue are: 0 0 15 25 26 17 0 0 14 0.

Then, if January 6^th^ is the target day, the predictor/predictant pair 5LC/LC6 is (0 0 15 25 26)/17, and its correspondent pair of binary vectors 5BLC/BLC6 is (1 0 0, 1 0 0, 0 −1 −1, −1 −1 −1, −1 0 −1)/(0 1 −1).

We have used CWT data of different periods, the largest of 20 years between 1975 and 1994, with 7300 days. The inhomogeneous distribution of LCs over London (UK) showed in Table 1, can be now explored by analyzing how the observed BLC6 vectors fill the 3D vector space. Table 4 shows some estimators of the 3D BLC6 vector dispersion, calculated for three different sets, the 7300 vectors of 20 years, the 360 vectors of one year and, as a reference of completely dispersed set, a 7300 3D vector set of components +1, 0 or −1 generated at random. While the average components, <BLC>, for the random set are almost zero, those of the observed wind vectors show the expected bias, low bias to A and S and high bias to W. The rest of the average estimators described in the Table 4 (<σ>, <dist> and <α>) confirms the less dispersion of the observed data with respect to a random vector set.

**Table 4:** Estimation of the CWT data distribution having into account their BLC codified vector dispersion on 3D space.

N (days)	<BLC>	<σ>	<dist>	<α>
7300 (random)	0.00,−0.01,−0.00	0.82	1.87	90.01
7300 (75_94)	0.09,−0.09,−0.29	0.69	1.56	86.57
360 (1995)	0.12,−0.04,−0.23	0.70	1.57	87.64

First row for a randomly generated BLC set of vectors, second and third rows for observed BLC data. In the second column are the components of the average vector <BLC>, in the third column are the averages of the correspondent root mean squares deviations <σ>, in the fourth column are the average distance between all pairs of vectors <dist>, and in the fifth column the average angle between all pairs of vectors <α>.

### Data classification by Kohonen Neural Networks (KNN)

In the section of METHODS we show in detail the KNN process. In the present section we just describe how KNN works with our CWT data. We first use KNN to project all the 15D 5BLC vectors of the considered period, with a similarity criterion among them, on points of a 2D Kohonen map. In this map, the vicinity among the projections, generally forming clusters, implies the similarity of their respective 15D vectors. This KNN process is called unsupervised training. A supervised training is the projection the (15+3)D union vectors formed by the 15 components of 5BLC and the 3 components of the correspondent BLC6 vectors, by considering the total similarity in between.

In an unsupervised training, the KNN allows simultaneous memorization of all input 15D 5BLC vectors in a 3D learning matrix. After some iterations, or training epochs, 15D vector-images, similar to the input vectors, cluster in this matrix. When the training converges, a cluster analysis can be done on the vector-images projected on the 2D Kohonen map. To identify each vector-image on the Kohonen map, we label each one by the LC6 observed after the correspondent 5BLC input vector. In this way, if equal LC6 labels cluster on the 2D map we guess that some correlation exists between 5BLC and their BLC6 classes. In fact, we found this correlation in our CWT data, which confirms that KNN is useful to uncover that the 5BLC vectors contain some information of their correspondent LC6.

In a supervised training, the 3D learning matrix increases its size to contain the vector-images of the (15+3)D 5BLC/BLC6 input vectors, this matrix will be later used for prediction purpose. Once the 5BLC vs. BLC6 correlation is confirmed, it is justified to have the BLC6 information into account for the 5BLC classification, which gives a more resolved Kohonen map. The supervised-trained 3D matrix and its correspondent Kohonen map, give then information about the most probable CWT sequences before each one of the 27 CWT's. The probable CWT sequences are the 15D imaging vector in the matrix corresponding to the desired LC6 type on the Kohonen map.

This 5BLC/BLC6 correlation implicit in the 3D trained matrix for a large period, can be used to predict the CWT of one target day from the CWT sequence of the previous five days, for a different test period (see in METHODS). For this, we have to assume, and in fact we prove, that similar CWT time series of the test period can also be found during the training period. Note that, in order to increase the probability of this event, we take a long training period of twenty years before to the one year test period. The prediction is done by looking for the image vector most similar to our predictor 5BLC sequence in the predictor part of supervised-trained 3D matrix (the distance between both 15D vectors is the localization error *δ_loc)*. Then, the correspondent predictant part of the vector is the predicted CWT for the target day (the distance between this 3D predictant vector and the actual observed BLC6 vector is the prediction error *δ_pred*).

**Figure 2:**
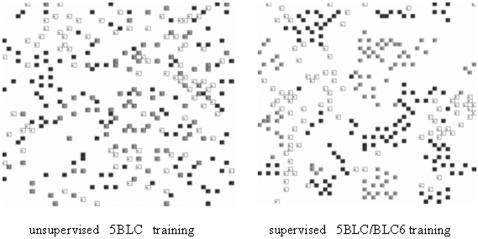
The 50×50 Kohonen maps of the unsupervised clustering (left) and of the supervised clustering (right) of the 5BLC vectors during 1992–93. For clarity reason, only the labels of the most populated LC classes A (black), C (grey) and W (white), are shown. Self-aggregations of equal LC6 labels are amplified by supervising. A given LC spreads on some clusters each one corresponding to a cluster of similar 5LC sequences.

We have seen however that the clustering of 15D-5BLC vectors in the unsupervised Kohonen map or the clustering of 18D-5BLC/BLC6 vectors in the supervised map, are far from perfect if we expected a map just with 27 separate clusters for the 27 classes of LC6. We have instead overlapped clusters indicating a diffuse 5BLC/BLC6 correlation, although we realized that some significant clustering and hence correlation exist. The Figure 2 shows the Kohonen maps, one unsupervised and the other supervised for the 726 5BLC/BLC6 vectors during 1992–93 after 4000 training epochs. Both maps are 2D-periodic and their cluster positions cannot be related as they correspond to two independent trainings. The maps are 50×50 size which give 3.4 points per input data and for clarity we only show the labels of the most populated LC's (A = 0, C = 20 and W = 16).

The maps don't show random distributions but rather we see several diffused clusters per class with better resolution in the supervised map. However, the maps indicate that similar 5BLC vectors in a cluster can precede different LC6 or that one LC6 can be preceded by different 5BLC clusters. Both effects can be reduced by smoothing the trained matrix which will also smooth the LC distribution on the Kohonen map (see in METHODS). In the same way, in order to find in the map the most representative clusters of a LC6, it is useful to calculate for each LC its density map d(i,j). This is done by counting at each point (i,j) the number of LC's of this class in an area around the point (also including the overlapped LC6's) in the Kohonen map.

**Figure 3:**
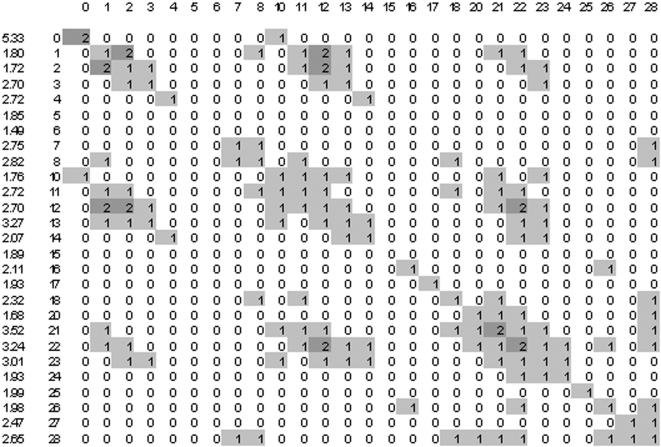
Neighbourhood matrix of LC6 classes, with rounded values of VN3(i,j)×100, for the non supervised Kohonen map in the period 1975–94, where W has been smoothed three times. The non-zero values are coloured to show the closer classes on the map. In the first column are the aggregation factors for each LC, in the second column the LC6 numeration of Table 3.

**Figure 4:**
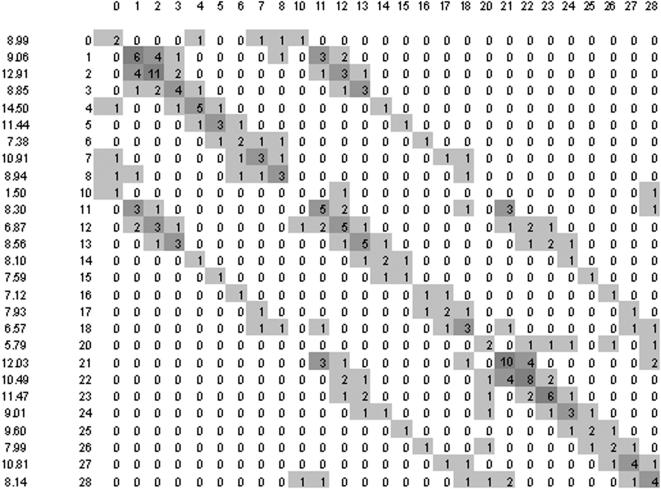
Neighbourhood matrix of LC6 classes, calculated as that of Figure 3, but for the Kohonen map after supervising the training with the CWT of the 6^th^ days.

The diffuse correlation between 5BLC and LC6 classes hinder a direct interpretation of the Kohonen map in terms to evaluate the self aggregation of LC6 classes in clusters, with the parallel aggregation of their correspondent 5BLC classes. In fact, the more defined is the 5BLC/LC6 correlation, the more defined becomes the self aggregation of each LC6 in terms of few isolated and compact clusters. A measure of these cluster properties can be done in the 27×27 normalized neighbourhood matrix VN3(i,j) among all LC6's on the 2D map (see Figures 3 and 4 for matrices of unsupervised and supervised trainings with 20 years data). We show in METHODS how the matrix is calculated and how an aggregation factor can be defined for each LC6, which should be greater than one for a significant LC6 aggregation.

### 5BLC/BLC6 vector correlation

Although the contribution of the 3D-BLC6 vectors in the supervised training of the learning matrix W is only 1/6 of the input vectors 18D−(5BLC+BLC6), it will produce some bias in the classification. Therefore, in order to prevent undesired artificial bias, it is necessary to ensure that significant correlation exists between both vector sets 5BLC and BLC6 before proceeding with the supervised training. On the other hand if that correlation exists, it would be justified to supervise the training in order to amplify that correlation, which will allow a further improvement on the LC6 prediction.

We show in this section several evidences supporting 5BLC/BLC6 correlation. The first one, shown in the previous section, is that the 15D 5BLC vectors of 1992–93 are self-classified, after unsupervised training, by their corresponding LC6 labels. Although the correlation is diffuse, the aggregation factors for LC = (30, 20, 16), in the Kohonen map of Figure 2, are respectively (2.3, 1.5 1.9) and their average value for all 27 classes is 2.4, being these values significantly greater than 1 (the average aggregation factor for a random distribution of classes on the map). The second evidence is that the above training for 1992_93 supervised by their BLC6 vectors amplifies that implicit correlation, as shows Figure 2, improving the above aggregation factors to (4.5, 3.3, 4.8) with average of 4.0, double values although the supervising only modifies 1/6 of the training vectors.

On the other hand, the 5BLC/BLC6 correlation can be generalized to other periods. Thus, prediction of the 1994 BLC6 vectors from their previous 5BLC, by using the supervised W matrix with 1992_93, gave an average <*δ_pred>* = 1.25, which decreases to 1.11 by smoothing the matrix indicating that it represents better the 1994 test period. Both errors indicate correlation since they are significantly lower than the average error of 1.54 for a BLC6 prediction by using an untrained or randomly generated W matrix. This random prediction error is almost constant for different random matrices or different BLC6 sets, so we used it to validate prediction errors from trained matrices.

As another evidence for 5BLC/BLC6 correlation we did the following test. It is expected that the supervised training would only be effective when the 5BLC vectors contain significant information of their correspondent BLC6 vectors. We confirmed this by supervising the 5BLC's wind sequences of 1992_93 with the non correlated winds of the 36^th^ day ahead, BLC36. As expected, the lack of correlation prevented the prediction of BLC36's corresponding to 5BLC's of 1994, giving <*δ_pred>* = 1.56 (close to the error for a random prediction). Using that ill-biased matrix, the BLC6 corresponding to the 5BLC of 1994 could neither be predicted, giving <*δ_pred>* = 1.55, which also confirms what it was expected.

In addition, we found that the increasing of the aggregation factors in the Kohonen map, from unsupervised to supervised training, is much more relevant for longer periods. Figures 3 and 4 show the LC6 neighbourhood matrices for the non-supervised and supervised Kohonen maps, for the period 1975–94 with 7300 input data. In both cases the learned matrices have been smoothed (see in METHODS) three times in order to minimize local singularities. We don't show the Kohonen map due to its 162×162 big size necessary to conserve ∼3.5 neurons per input data. Figures 3 and 4 also give the individual aggregation factor for each LC, and we note the large influence of supervising on the average aggregation factor, which increases from 2.46 to 8.92 (2.41 to 8.55 by using unsmoothed matrices), twice the effect compared with the 1993–94 training. In fact, 7300 5BLC input data represent 10 times better the possible series of 1995, than the 726 input data of 1992–93 the series of 1994. Note that the possible combinations of the 27 LC's among 5 days is quite big (27H5≈170000), although many combinations are very improbable.

Besides the LC aggregation factors, the neighbourhood matrix gives more local valuable information. If we colour for growing values the elements of the matrix, we get a repetitive pattern in both Figures 3 and 4. The values on the main diagonal VN3(i,i) show the aggregation of equal LC6's, the values of VN3(i,j) with j close to i show the aggregation between different but close BLC's having the same vorticity (the proximity should be interpreted on the winds cube). In addition there are other parallel diagonals of significant values at periods of 8 LC's showing the aggregation of also close LC's in the winds cube but with different vorticity. According to the aggregation factors, the rather diffuse neighbourhood matrix of the unsupervised training becomes sharper by supervising the training, which amplifies the correlation between closer CWT's. For example, the supervised training doesn't cluster together A with C winds, whereas the unsupervised training does.

The relative very low aggregation factor of 1.5 in the supervised training, corresponds to the 74 days, along 1975–94, with LC = 10 or total undefined (UND) CWT (0,0,0), which cannot be properly normalized with KNN.

### Probable wind sequence preceding a given wind class

Choosing a large training period will include more 5BLC/BLC6 observed samples and, as we showed before, it will also increase the aggregation factor from unsupervised to supervised Kohonen maps. Hence, in order to analyze the most probable sequences of five winds preceding a particular class of wind for the 6^th^ day, we have used the supervised trained matrix (smoothed 3 times) and its 162×162 Kohonen map, with 1975–94 wind data and 10000 epochs, which corresponds to the neighbourhood matrix of Figure 4. As we also showed before, there are some reasons to choose the smoothed matrix. First that it increases the aggregation factors of both unsupervised and supervised maps, which means that the smoothed matrix represents better the correlation between the 5BLC/BLC6 training vectors. Second, that the smoothed matrix of the 1992–93 training data represents better the 1994 test data than the unsmoothed one as it gives better prediction, which was also confirmed for the periods 1975–94/1995.

The most significant clusters of each LC6 on the 162×162 Kohonen map, are found on their particular density map up to 5 neighbours (defined above in Data Classification by KNN), where we choose the three largest maxima. The centre of each density maximum corresponds in the smoothed matrix to the most representative wind sequence for the 5 days previous to that LC6 class. The components of this 15D vector are then approached to integer values 1, 0 or −1, to identify the closest 5BLC, which together with the LC6 are shown in Table 5. Of course, the significance of the wind sequences previous to each LC6 depends on their population in the training period, and consequently on their BLC6 adjustment to the matrix, both listed in Table 6.

**Table 5:** The three most probable sequences of five daily winds, preceding each LC wind class.

A	A	A	A	A	A,	AW	A	A	A	A,	ASW	SW	AW	ANW	AS
ANE	A	A	A	A	AE,	A	A	SE	E	A,	W	CNW	C	CNE	NE
AE	A	A	A	A	A,	AE	ANE	E	E	NE,	E	E	E	A	NE
ASE	A	S	AS	AS	ASE,	ASE	SE	SE	ASE	ASE,	S	A	ASE	SE	E
AS	A	A	A	A	A,	A	A	A	A	AS,	C	CS	C	C	W
ASW	C	C	C	CSW	CS,	ANW	ANW	ASW	C	CSW,	CSW	C	AW	CSW	SW
AW	CN	AW	CW	CW	AW,	A	W	AW	A	ASW,	S	S	CSW	SW	W
ANW	W	CW	W	C	NW,	AW	ANW	AW	NW	ANW,	AS	A	A	A	A
AN	A	SW	CSW	C	NE,	C	C	CNW	E	CNE,	A	A	A	A	N
UND	A	A	A	A	A,	NE	NE	N	UND	C,	CSW	W	C	C	NW
NE	A	A	AN	NE	NE,	W	SW	CNW	CNW	NE,	S	C	C	CNE	NE
E	E	NE	NE	AE	E,	A	A	A	AE	E,	SE	SE	SE	SE	SE
SE	A	S	SE	S	SE,	S	S	S	SE	SE,	SW	SW	ASW	A	S
S	S	S	S	CS	S,	AN	AS	S	S	S,	A	A	A	CS	ASW
SW	ASW	SW	W	CSW	NW,	W	W	SW	AW	ASW,	CW	W	SW	CSW	SW
W	W	CW	W	W	CW,	A	A	A	ANW	NW,	S	SW	W	S	SW
NW	SW	CSW	W	W	CW,	N	NW	ANW	NW	NW,	AS	ANE	A	A	NW
N	NW	NW	NW	NW	CNW,	NW	N	N	N	CN,	W	NW	W	C	CNW
C	C	C	C	C	C,	AW	C	C	CSW	CSE,	S	SW	C	C	CSW
CNE	ASW	SW	CSW	C	C,	E	E	UND	ANE	CSE,	C	C	C	C	CE
CE	SE	SE	SE	NE	CNE,	SE	SE	SE	E	CE,	CSE	C	C	CE	CE
CSE	SE	A	A	S	CSE,	S	CSE	C	CE	SE,	S	SE	SE	SE	CSE
CS	C	C	SW	S	CS,	CS	SW	S	C	CSW,	NE	AE	CN	CN	CS
CSW	ANW	SW	SW	SW	S,	AE	SE	C	C	C,	ASW	CSW	CS	C	SW
CW	CNW	SW	NW	W	W,	C	C	CS	CS	CW,	A	A	SW	SW	C
CNW	NE	CE	C	CS	C,	SW	W	CSW	NW	NW,	C	NW	NW	NW	W
CN	ANW	ANW	ANW	W	C,	CW	W	CW	W	C,	ANE	ANE	AN	N	NW

In the first column on the left are the 6^th^ day LC classes, then, the previous five wind sequences separated by commas, from the most (left) to the least probable sequence (right). For each sequence, from left to right the observed average winds in the 5th, 4th, 3rd, 2nd and 1^st^ previous days. These winds have been calculated from the smoothed learned matrix of a KNN, after a supervised training with the 7300 5BLC/BLC6 vector pairs in the period 1975_94.

**Table 6:** Distribution of the 27 LC's of table 3, in row A, among different periods, LC = 10 for completely undefined wind is omitted.

A	0	1	2	3	4	5	6	7	8	11	12	13	14	15	16	17	18	20	21	22	23	24	25	26	27	28
B	134	3	7	3	8	21	27	17	11	17	15	20	33	78	85	40	24	87	5	4	5	10	23	17	16	8
C1	1482	53	62	69	118	185	214	147	97	137	123	193	401	672	779	416	282	974	44	35	66	124	182	157	134	80
C2	0.28	0.89	0.61	0.86	0.67	0.73	0.65	0.79	0.71	0.69	0.55	0.54	0.52	0.44	0.39	0.52	0.53	0.31	0.84	0.69	0.84	0.71	0.75	0.66	0.77	0.68
D1	75	5	6	4	4	8	12	10	2	9	6	12	20	26	31	24	18	46	1	3	4	3	6	14	2	3
D2	0.80	1.58	1.04	1.86	1.63	1.36	1.10	1.39	1.60	1.32	0.98	1.50	1.05	1.16	0.88	1.34	1.15	1.03	1.16	1.59	2.03	0.68	1.77	1.07	1.34	1.23
D3	0.71	1.30	0.96	1.80	1.25	1.32	1.17	1.55	1.86	1.03	1.01	1.29	0.94	1.19	0.88	1.08	1.12	0.77	1.01	1.31	1.83	1.41	1.41	1.19	1.38	1.07

In row B the LC distribution in 1992–93. In row C1 the LC distribution in 1975–94, and in C2 the average ‘prediction’ errors for LC6 vectors after a supervised training. In row D1 the LC distribution in 1995, in D2 the average prediction errors for LC6 vectors after supervised (with the 6th day) training of 1975–94, and in D3 after supervised (with the 5th day) training. All errors have been calculated with the smoothed matrices.

As expected, considerable wind persistence is observed in Table 5, and it is important to remember here that the classes A and C, with strong wind persistence, can be quite biased as a result of considering daily average winds.

It is also important to note that the wind sequences of Table 5 are not necessarily observed sequences in 1975–94. In fact they were initially their image vectors (close to the observed sequences) but then were averaged by smoothing and later approximated to integer values to the closest observed wind sequence. In any case, Table 5 gives the most probable wind sequences previous to a given LC, considering the 1975–94 period. Besides, we will see in the next section that the localization errors of the five wind sequences among the test set of 1995 or among the training set of1975–94, on the smoothed matrix trained with 1975–94, are similar (see Table 7), indicating that this matrix represents both periods. This fact, emphasize the impact of the training set size on the prediction of the test set. Hence, Table 5 could be generalized for other periods, such as 1995, not included in the training.

**Table 7:** Wind (BLC6) prediction from the previous (5BLC) five days data wind by Kohonen Neural Networks.

	sup	sx	coin	δV0	nw-w	δα	δV1	nw-w	δα	δV2	nw-w	δα	<*δ_loc*>	<*δ_pred*>	*<δ_pred* >_1_
75–94	6	0	7136	7180	26	0.1	46	0	1	0			0.29	0.09	0.01
	6	3	4769	6365	713	7	861	77	12	0			1.22	0.48	0.37
1995	6	0	62	157	47	33	162	79	50	35	17	45	0.98	1.24	1.30
	6	3	70	168	35	33	155	69	50	31	15	49	1.29	1.10	1.24
1995	5	0	106	189	37	19	147	70	33	18	4	30	0.97	0.98	1.00
	5	3	92	175	35	21	159	65	33	20	9	28	1.27	1.01	1.06

After the row of variable labels, the first and second rows are for the KNN training along 10000 epochs, in the learning period 1975–94 (7300 days), supervised by the winds of the 6^th^ days. The third and fourth rows are for the 6^th^ day wind (real) prediction in 1995 by the above training. The fifth and sixth rows are for the 6^th^ day wind (real) prediction in 1995 by similar training but supervised by the winds of the 5^th^ days. The supervising day is indicated by *sup, sx* is the times the learned matrix has been smoothed with sm = 2/3, *coin* is the number of total (wind direction plus vorticity) coincidences between observed and predicted winds. Next columns to the right are, the first set (δV0 nw-w δα) for the number of days, δV0, having the same vorticity for the observed and the predicted winds, the second set when the vorticity difference is 1, δV1, and the third set when the difference is 2, δV2, (A to C or C to A). The other two columns of each set are the number of days changing from no-wind to wind (nw-w), or vice versa, and the average angle between the observed and predicted winds (δα). The last three columns are the average localization error for the 5BLC vectors <*δ_loc*>, the prediction error for the BLC6 vectors <*δ_pred*>, and the same error but approaching the matrix components to 1, 0 or −1, to get the closest possible observed vector *<δ_pred* >_1_.

It is also important to note that the 5 days CWT time series, shown in Table 5, are just the result of an objective self-clustering of all observed 5-days time series along 1975–94 together with their observed CWT in the 6^th^ day. In fact, the lack of meteorological assumptions to derive Table 5, showing purely statistical analysis of the observed data, makes it valuable for its posterior meteorological interpretation or for testing a prediction model. In fact, it is known that atmospheric circulations are patterns repeated over periods of time and hence they are often predictable.

Some analysis could be done on Table 5. For example, it is interesting to show the vorticity persistence, that is, the distribution of vorticities types among the 5 days previous to each vorticity type of the 6^th^ day. Thus, previous to A there are 73A, 29C and 33UNDef., previous to C 17A, 65C and 53UNDef., and previous to undefined vorticity there are 33A, 22C and 80UNDef.

A second analysis of Table 5 would be to identify the time series on the winds cube, giving a 3D graphical representation of the circulation variation among these five days.

A third more difficult analysis of Table 5 would be to get, from the evolution among five days of the CWT in a point (London), some information about the 2D circulation map evolution responsible of that CWT time series. For a point in the Northern Hemisphere the winds rotate around an Anticyclone in a clockwise manner, while the rotation is counter clockwise around a Cyclone. Then, if we assume the simple case of circular shaped Anticyclone or Cyclone causing wind on a point P, from the direction of this wind one could estimate the situation of the centres of these A or C, with respect to P, as shows the first part of Table 8. Now, as an example, let us take from Table 5 the most probable wind sequence, from the fifth to the first day before the target day with AN: A SW CSW C NE. Then, according to Table 8, the situation of the centres of A and/or C, with respect to P, would be for the 5^th^ day before (A) an A centred on P, for the 4^th^ day before (SW) an A centred to the SE of P and a C centred to the NW of P, and so on as it is shown in the second part of Table 8, which shows a continuous displacement of A and C, across P, towards SE direction.

**Table 8:** Above, the probable situation of an Anticyclone or Cyclone, with respect to a point P receiving their wind. Below, as an example, the probable evolution of the 2D circulation map previous to a day with CWT = AN

Wind on P coming from	N	NE	E	SE	S	SW	W	NW
A situation with respect to P	W	NW	N	NE	E	SE	S	SW
C situation with respect to P	E	SE	S	SW	W	NW	N	NE
Day before	5	4	3	2	1	0		
Situation of A or C with respect to P	A(on)	A(SE) C(NW)	C(NW)	C(on)	A(NW) C(SE)	A(W)		

### Prediction of wind direction and vorticity for a given day from wind data of the previous five days

We select for prediction purpose the 20 years (1975–94) large training period because the aggregation of CWT's in the Kohonen map shows higher correlation between 5BLC/BLC6 input pairs, and because the CWT time series of the next year, taken as test period, are well represented in that training period. After 10000 epochs of supervised training with the period 1975–94, the 5BLC/BLC6 input vectors are adjusted to their correspondent vector images in the matrix, as shows Table 7 under <*δ_loc*> and <*δ_pred*> columns, which obviously get worse by smoothing the matrix since the process adjusted the unsmoothed matrix. Note that in this case both localization and prediction mean just adjustments. The Table 7 also shows observed/predicted wind coincidences, errors in vorticity and angle errors in wind directions. The unsupervised learning matrix with 1975–94, which is not in Table 7, gives a 5BLC localization error of 0.22, lower than that of supervised learning, as expected. Table 6 shows the number of days per class and the <*δ_pred>* adjustment per class, which is better for the most populated classes. Of course, these “prediction errors” apply to the most probable wind sequences previous to each LC shown in Table 5, as both deal with the same smoothed trained matrix.

The supervised trained matrix is then used for a real BLC6 prediction from their previous 5BLC, but in a test period (1995) not used in the training. Now, the generalization of the learning to a different period is tested by comparing the known BCL6 winds of 1995 with the predicted ones. For this process, we are assuming both, that the 1995 5BLC vectors are represented enough in the learned matrix with 1975–94, and that there is similar 5BLC/BLC6 correlation in both periods. In fact, Table 6 shows that the distribution of CWT's during 1995 is similar to that for 1992–93 and 1975–94. It also shows that the prediction errors per class are lowest for the most populated classes. The total UNDefined winds have been omitted of Table 6 and 7 (74 in 1975–94 and 6 in 1995) as they cannot be well processed by KNN.

After the analysis of the 1975–94 training period, Table 7 shows the localization and (real) prediction errors for the CWT of 1995 together with vorticity and angle partial errors. As we said before, the similar localization error of both five wind sequences in the smoothed matrix, 1.22 for 1975–94 and 1.29 for 1995, indicates that this matrix represents both periods similarly. As expected, the BLC6 real prediction error of 1.10 for 1995 is higher than the adjustment error of 0.48 for the BLC6 of 1975–94, as the later were included in the supervised training.

In order to test the wind persistence, we calculated again the learning matrix with 1975–94, but this time supervising with the 5th day wind BLC5, instead of with the 6th day wind BLC6. The result is not in Table 7, but it gives a localization error of 0.22 and an average “prediction” error of 0.03 for BLC5's, both smaller than supervising with the 6^th^ day. By using the corresponding trained matrix, the prediction error for the actual 6th days in 1975–94 is 1.00, which in fact is close to the average distance among all (7300) observed BLC6 and their previous observed BLC5 (that is the prediction error assuming persistence), due to the high fit of 0.03 or almost coincidence of BLC5's to the matrix. The Table 7 shows that the supervised matrix with the 5^th^ days is able to predict the winds of the 6th days of 1995 with a similar average prediction error of 0.98, slightly higher than the average error of 0.967 between the observed BLC5 and observed BLC6 in 1995. This proves again that the 5BLC of 1995 are well represented in the 1975–94 matrix and that wind persistence is similar in both periods. The prediction error in this case doesn't improve by smoothing the matrix. Table 6 also shows the distribution errors of predicted BLC6 for that training supervised with the 5^th^ day winds.

Hence, just by assuming CWT persistence from one day to the next gives a bit lower average prediction error than by using a KNN trained matrix with 5BLC/BLC6 data of a large period. By supervising with the 5^th^ days we reinforce its influence on the training giving similar prediction as persistence. However, although the observed wind persistence is clearly conditioning the average prediction, the data mining by KNN allows individual insight into the wind sequences distribution and the particular wind prediction from the previous wind sequences, beyond the average results, which can be clearly seen in Tables 5 and 6. On the other hand, there are 246 days in 1995 with different wind as the day before, for which we get <*δ_pred>* = 1.32 and there are 176 days with different wind to any one of the five previous days, for which we get <*δ_pred>* = 1.36 (both lower than 1.54 for random prediction). This confirms that there is some hidden CL6 information included in the previous 5BLC aside persistence. However, the principal contribution of the KNN approach to CWT prediction is that, contrary to the standard methods, it just depends of an objective classification of observed CWT data without any other assumption.

By using the 5BLC/BLC6 training we got an average error for predicted wind directions of 41° (after averaging δα of the fourth row in Table 7). This is the same error as obtained by Ultsch and Röske [13], by using KNN with the North Sea wind data plus pressure and temperature data and shorter interval between measurements; with these data, they got a wind direction error of 38° by assuming wind persistence.

The 5BLC/BLC5 training (fifth row of Table 5), which gave similar results as to assume LC5/LC6 persistence, gives an average error for wind direction of 24°, equal to that given by a conventional wind forecast model in the mentioned South Eastern part of the North Sea, provided by the Marine Weather Service in Hamburg, cited as SWA by Ultsch and Röske [13].

## Discussion

We used the Lamb Catalogue for Circulation Weather Type (CWT) data including daily average of wind direction and its vorticity over London, UK. The 27 Lamb Classes (LC) where codified in 3D binary vectors pointing to vertex, edges and faces of a winds cube, which gives similar close vectors for similar LC's.

Data mining of those codified LC's by Kohonen Neural Networks (KNN) allows self-classification, or objective clustering, of 5LC time series along five consecutive days. In this classification, the correspondent observed LC6 for the sixth day (not included in that classification) become also somehow classified among the 27 LC parallel classes. This means that the 5LC wind series conditions somehow the wind of the last LC6 day, and that the explicit correlation by wind persistence and other unknown implicit correlation are uncovered by the KNN data mining. The choice of five days for time series was suggested by the observed border of persistence decay over five days.

The 5LC/LC6 correlation allows giving the most probable wind sequences previous to each one of the 27 LC's, in the long KNN trained period of 1975–94, or by generalization, in a different not trained test period like 1995. Inversely, it also allows predicting the probable CWT of a day in the test period, by knowing the precedent CWT sequence. The generalization is possible because the wind sequences of the test periods are enough represented in the training period. This presence of similar wind sequences along different periods causes the KNN prediction skills, the longest the training period the better, although we could also find enough representation of 1994 time series just in the 1992–93 period.

Although the observed wind persistence is an important part of the 5LC/LC6 correlation and strongly conditions the wind prediction, the KNN procedure also allows LC6 prediction for 5LC time series not including that LC6.

The correlation between 5LC classes and LC6 classes is however somehow diffuse, in fact the Kohonen map shows some overlapping between classes of the 5LC set, and their relation with the LC6 classes is far to be bi-univocal. The self classification of the 5LC data is analyzed by the aggregation of their correspondent LC6 on the Kohonen map. It is shown that this aggregation increases with the length of the training period, and the classes with high aggregation in the training period are the best predicted in the test period.

Our research shows results for a specific grid time and grid space, where the used daily average data can bias the real observations due to the high variability of the wind. As a result, the number of days with undefined wind direction or vorticity is probably overestimated. Although the computation time could be prohibitive, the use of longer data sets at higher resolution both in time and space would produce more accurate results.

The presented results are derived from an objective self-clustering of observed CWT time series without any weather model assumption; hence they just reflect what is observed after a mining process. This wind data mining analysis extracts and represents some facts and wind correlations hidden in the observed CWT data which, apart of its informative value, could be complementary for checking the calculated forecasting results with conventional forecasting models.

## Methods

### The Kohonen Neural Network

**Figure 5:**
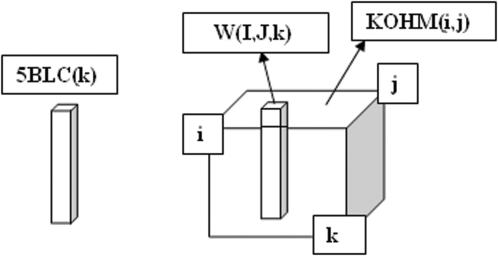
Scheme of the Kohonen NN. Each input vector 5BLC(k), on the left, activates every point of the 2D Kohonen map KOHM(i,j) by the 3D synapses matrix W(i,j,k). The values of W(i,j,k) are trained to cluster the input vectors in the self classified 2D Kohonen map.

Figure 5 shows a scheme of the Kohonen Neural Network, Kohonen [12]. Let us explain how it works with the following example. Assume we want to classify by similarity all the 726 (365+366−5) 5BLC(k) 15D vectors for all the 5 days wind sequences corresponding to the two year period 1992–93. The Kohonen NN is going to project each vector on a point of a 2D Kohonen map, which will be a matrix of 50×50 points (in order to have 3.4 points space for each input vector, as 50×50/726 = 3.4). To the left of Figure 5 there is a column with 15 neurons which will be activated by the components of the input vectors 5BLC(k)_L_, where L indicates learning. To the right is the cubic learning matrix (50×50×15) W(i,j,k) of the variable synapses (connection weight) between the 15 input neurons to each one of the 50×50 output neurons, which form the 2D Kohonen matrix represented on the superior face of the cube. The activation of each neuron of the Kohonen matrix is calculated by KOHM(i,j) = ∑_L_5BLC(k)_L_•W(i,j,k). At the beginning of the calculation, the 37500 (50×50×15) elements of W are fractional random values between −1 and 1. Then for each one of the 726 input vectors the training begins: the column vector W(I,J,k) most similar to it is searched, and modifications of the W(I,J,k) elements and (with less intensity) also of W(i,j,k) neighbour columns, are calculated (but not applied) to approximate them to the input vector 5BLC(k)_L_. All (50×50×15) synapses modifications due to the consecutive 726 input vectors are accumulated and then are applied to W(i,j,k), completing one epoch of training. Both, the maximum radius for neighbour W columns and the strength of synapses corrections, decay as the learning advances. After N epochs, the 3D synapses matrix is full trained if the components converge to stable values, and then each input vector 5BLC(k)_L_ will have its image, with similar components, in a particular column vector W(I_L_,J_L_,k). The process has given similar components to neighbour columns, establishing therefore a self-classification, by similarity, for the 726 input vectors. The vector images of similar input vectors cluster in the 3D matrix and are mapped on the Kohonen map at the points KOHM(I_L_,J_L_). Each 5BLC vector is identified on the map by a label, for which we use the correspondent observed LC6. For neighbourhood considerations the Kohonen map is built periodic in its two dimensions (all computing programs used in this work were made by us and are available upon request).

If some aggregation of equal LC6 labels into classes is observed on the map, it means that there is correlation between the 15D 5BLC vectors and their corresponding 3D BLC6. In this case, a classification of these 5BLC vectors together with the corresponding BLC6 vectors will be useful to amplify that correlation. This is a supervised training, where each input vector is formed by the union of the 15+3 components in an 18D vector, the learning matrix W(i,j,k) becomes now of dimension (50×50×18), and the calculation proceeds like the non-supervised training.

If the unsupervised training converges, the image column vectors W(I_L_,J_L_,k) become almost coincident with its corresponding 5BLC(k)_L_, and the same thing happens in the supervised training among the last three components of W(I_L_,J_L_,k_16–18_) with the corresponding components of BLC6(k)_L_. We call <*δ_loc*> (localization error) the averaged value of the distances among the learning vectors 5BLC(k)_L_ and their images W(I_L_,J_L_,k_1–15_), and <*δ_pred*> (‘prediction’ error) the averaged value among the distances between BLC6(k)_L_ and W(I_L_,J_L_,k_16–18_). Although in this case *δ_pred* is just an adjusting error as BLC6(k)_L_ were implied in the W learning.

Assuming deterministic rules for wind evolution, or that similar wind time series can be found in different periods, the knowledge of a training period could be used to predict winds in a different test period. Thus, the learned matrix W(i,j,k) obtained by a supervised training with data from 1992_93, can be used to predict, for the test year 1994, the 360 3D wind vectors, BLC6_T_, from their corresponding 15D wind vectors 5BLC_T_ of the previous five days. To do this, for a given 5BLC_T_(k) vector, we look in the above W(i,j,k) for the more similar column vector W(I_L_,J_L_,k_1–15_) and then, the last three components W(I_L_,J_L_,k_16–18_) of that column will be the components of the corresponding predicted vector for the observed BLC6_T_ in 1994 (by using our 1992_93 data knowledge). Thus, the predicted LC6_T_ will usually be, on the Kohonen map, close or included into a previous self-classified cluster of similar LC6_L_ classes. The average localization error for 5BLC_T_ shows how the test data are represented in the learned matrix. The ‘prediction’ errors are now real prediction since the test period was not included in the matrix training.

### The learning conditions of the KNN

In the learning process itself, there are some control parameters such as the number of neurons in the Kohonen map (*numK*) or its size, and the optimal number of learning epochs (*numE*). For example, Ultsch and Röske [13] recommended *numK* = 10×*numV* and *numE* = 10×√*numV*, where *numV* is the number of training vectors. But we didn't use that control since we have a large *numV* = 7300 for 20 years, which would produce a too long calculation. We decided instead to use a 162×162 map, allowing 3.4 neurons per learning vector and we needed *numE* = 10000 epochs to reach convergence in the learning matrix.

Another fact to consider is the possibility to have over-fitting during the training, this occurs when due to an excess of epochs, the net loses its generalization capability. Over-fitting is detected when the prediction error for test vectors (not included in the training) stops decreasing during the epochs, while the error for the learning vectors still decreases. In our case we could not observe any over-fitting along the different trainings.

### Smoothing matrix W

In order to minimize the local singularities on the Kohonen map, the learning matrix can be smoothed to obtain a Kohonen map with the most representative clusters. The smoothed WS(i,j,k) matrix is obtained by averaging each k column of W(i,j,k) with their neighbouring columns as follows: WS(I,J,k) = [16*sm* ^2^<W(I”,J”,k)>+8*sm*<W(I′,J′,k)>+W(I,J,k)]/(16*sm* ^2^+8*sm*+1), where WS(I,J,k) is a column vector in the smoothed matrix, W(I,J,k) is the column in the trained matrix, <W(I′,J′,k)> is the average of the first 8 neighbour columns around, <W(I”,J”,k)> is the average of the 16 second neighbour columns, and *sm* is a weight factor. We tried different *sm* values and selected *sm* = 2/3 as more convenient.

### Neighbourhood matrix

We found that a way of analyzing the clusters of LC6 classes on the Kohonen map is by calculating its 27×27 neighbourhood matrix V(i,j). For all points of the map occupied by a particular LC6 = i, we add in V(i,i) their neighbours with the same LC6, and in V(i,j) their neighbours with different LC6 = j, keeping in mind the periodicity of the map. This way, for a given vicinity radius on the map (at radius = 1 there are 8 points), a symmetrical neighbourhood matrix V(i,j) is calculated. However, as the number N_i_ of days for each LC class is different, we normalize the matrix to VN(i,j) = V(i,j)/(Ni×Nj). Then, we consider the value VN(i,i)/<VN(i,j)> for each LC6, as its aggregation measure on the map for a given vicinity radius (although may be LC6's spread on different clusters). Values of VN(i,i)/<VN(i,j)> greater than one means significant aggregation, hence significant self-classification for that LC6. Finally, for a better weight of the aggregation measure, we have accumulated in VN3(i,j), 3 times the first neighbours, 2 times the seconds and 1 time the thirds, then, VN3(i,i)/<VN3(i,j)> was the aggregation factor used in this work.
